# Effects of pore size and porosity on cytocompatibility and osteogenic differentiation of porous titanium

**DOI:** 10.1007/s10856-021-06548-0

**Published:** 2021-06-14

**Authors:** Yi-tong Yao, Yue Yang, Qi Ye, Shan-shan Cao, Xin-ping Zhang, Ke Zhao, Yutao Jian

**Affiliations:** 1 Hospital of Stomatology, Guanghua School of Stomatology, Sun Yat-sen University, Guangdong Provincial Key Laboratory of Stomatology, Guangzhou, China; 2 Department of Stomatology, Shenzhen People’s Hospital (Second Clinical Medical School of Jinan University; First Affiliated Hospital of Southern University of Science and Technology), Shenzhen, China; 3 Shenzhen Baoan Women’s and Children’s Hospital, Jinan University, Shenzhen, China; 4 School of Materials Science and Engineering, South China University of Technology, Guangzhou, China; 5 Institute of Stomatological Research, Guangdong Provincial Key Laboratory of Stomatology, Sun Yat-sen University, Guangzhou, China

## Abstract

To find out the optimal porosity and pore size of porous titanium (Ti) regarding the cytocompatibility and osteogenic differentiation. Six groups of porous Ti samples with different porosities and pore sizes were fabricated by the powder metallurgy process. The microstructure and compressive mechanical properties were characterized. The cytocompatibility was examined by a series of biological tests as protein absorption with BCA assay kit, cell attachment with laser scanning confocal microscopy and vinculin expression, cell proliferation with CCK-8 assay. Cell differentiation and calcification were detected by qPCR and Alizarin Red S dying respectively. Pores distributed homogeneously throughout the porous Ti samples. The compressive test results showed that Young’s modulus ranged from 2.80 ± 0.03 GPa to 5.43 ± 0.34 GPa and the compressive strength increased from 112.4 ± 3.6 MPa to 231.1 ± 9.4 MPa. Porous Ti with high porosity (53.3 ± 1.2%) and small pore size (191.6 ± 3.7 μm) adsorbed more proteins. More MC3T3-E1 cells adhered onto dense Ti samples than onto any other porous ones already after culture and no difference was identified within the porous groups. The porous structure of porous Ti with a porosity of 53.3 ± 1.2% and an average pore size of 191.6 ± 3.7 μm facilitated cell differentiation and calcification. Small pores were not beneficial to the osteo-initiation at the very beginning. Porous Ti with a porosity of 53.3 ± 1.2% and an average pore size of 191.6 ± 3.7 μm fabricated by powder metallurgy process showed the expected mechanical property and improved osseointegration as implants in dental treatment.

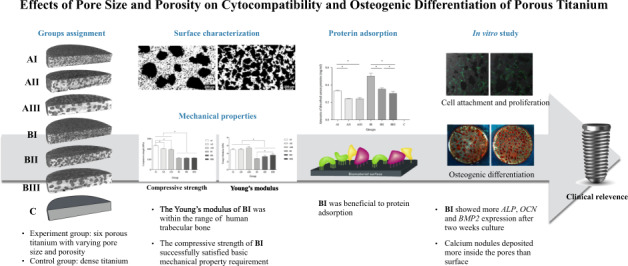

## Introduction

Titanium (Ti) possesses unique biocompatibility and has been widely used as an implant in dental treatment in the last few decades. In general, commercial Ti implants are mostly dense, with Young’s modulus (114 GPa) much higher than that of cancellous and cortical bone [1]. High modulus of the implant is known to cause stress-shielding at the bone-implant interface [2], and a dense superficial layer prevents the bone from ingrowing and forming interlock between the surrounding bone tissues, subsequently resulting in implant loosening. Porous structure was introduced to lower elastic modulus so as to match well with bone tissues, restrain bone atrophy and improve bone remodeling [3, 4].

In the fabrication of porous materials, porosity and pore size are the main twin factors. It has been reported that porosity of 66.1% showed best bone contact [5], whereas in another study porosity of 30–40% was suggested to have a positive effect on osteogenic differentiation and bone ingrowth [6]. It is well known that higher porosity facilitates bone ingrowth, but also weakens mechanical properties of the implant such as hardness, compressive strength, and elastic modulus at the same time [7].

In literature, the discussion of pore size remains no consensus. It has been suggested that the optimal pore size for bone ingrowth should be 100–400 μm [8–12]. Yet some studies found 600 μm of pore size might conduce to cell response and showed rapid bone ingrowth respectively [13, 14]. In other studies, pore size ranging from 284 to 416 μm was deemed to not affect cell proliferation and calcification [15].

Since porosity and pore size are inseparable twin factors, both can affect new bone formation and bone ingrowth [16, 17]. Study on either one of them will lead to distorting the understanding of both factors on their coordinated biological effect. Yet the related studies about these two factors were not directly comparable due to the different test materials. The biocompatibility and osteogenic effects provided by the material should be based on the material itself first since many more factors are also different besides porosity and pore size. In the present study, the optimal porosity and pore size will thereby be defined in porous Ti implants with respect to osteogenic differentiation and bone ingrowth.

## Materials and methods

### Porous Ti disks preparation

Six types of porous Ti samples with different porosities and pore sizes as Group AI to AIII and BI to BIII were fabricated by powder metallurgy process and sintered using conventional method [18]. NH_4_HCO_3_ powder was used as a temporary space-holder and pore size regulator. Commercial pure dense Ti (TA2) plates were served as controls (Group C).

### Microstructure and porosity

Samples were observed under metalloscope (Axio Image M2m, Zeiss, Jena, Germany). The microstructure was examined using a commercial micro-CT system (µCT50, Scanco, Bassersdorf, Switzerland). The protocols were standardized at 90 kV, 150 µA and 14 W with an integration time of 1500 ms for a singe of the two averaged scans per step and a filter of 0.5 mm aluminum. The voxel size was 14.8 μm and the scanning resolution was set at 2048 × 2048 pixels. The raw data set of each cross-section was converted to *dicom* format and 3D reconstructed in MeVisLab 2.1 to provide an axial perspective of the sample.

The general porosity (*p*) of the porous sample was calculated by the following Eq. (2-1):

2-1 $$p = \left( {1 - \frac{\rho }{{\rho _0}}} \right) \times 100{\mathrm{\% }}$$

where *ρ* is the apparent density of the porous Ti (measured by dividing the weight by the volume of the sample) and *ρ*_0_ represents the theoretical density of the corresponding dense Ti (4.51 g/cm^3^). *p* was also calculated based upon the measurement of the porous sample. The mean pore size (MPS) was auto-analyzed using Image-Pro plus.

### Mechanical evaluations

To demonstrate the mechanical properties, porous Ti with four specimens in each group were measured using a computer-controlled universal testing machine (AG-X, Shimadzu, Japan). Cylinder samples (10 mm × 10 mm, *ϕ* × h) were tested with a loading rate of 1 mm/min at room temperature (25 °C). Compression strength and Young’s modulus were calculated from the stress-strain curve of each sample.

### Protein adsorption

All groups of porous Ti disks with five samples per group were placed in 24-well plates respectively and each well was supplemented with 1 mL minimal essential medium (α-MEM) (Gibco, Grand Island, US) with 20% fetal bovine serum (HyClone, Thermo, Waltham, MA, US), 1% penicillin and streptomycin. Samples were incubated at 37 °C for 2 h under humidified 5% CO_2_ atmosphere. Samples were then washed in PBS thrice (5 min per wash) and transferred further to new plates containing 200 μL/well of 0.1% Triton-X 100 (Sigma, St Louis, MO, US) in 1× PBS, and incubated at 4 °C overnight. The total amount of proteins adsorbed was analyzed quantitatively using a commercial BCA Protein Assay Kit (Pierce, Thermo) and the absorbance was read at 562 nm by a microplate reader (Tecan, Männedorf, Switzerland).

### Cell culture experiments

The MC3T3-E1 cell line from the Cell Bank of China Scientific Academy (Shanghai, China) was expanded in α-MEM supplemented with 10% fetal bovine serum containing 1% penicillin and streptomycin. Cells were incubated at 37 °C under humidified 5% CO_2_ for appropriate time intervals. The growth media was changed every 2 days. Cultured cells were harvested at 70-90% confluence by trypsin-EDTA 0.25% (Sigma) and suspended in fresh culture media in readiness for the following experiments.

#### Cell attachment

Cells were seeded at a density of 2 × 10^4^ cells/well in 24-well plates. Complete culture medium and standard culture conditions were employed. After 1 day and 3 days incubation, cell adhesion was observed under a laser scanning confocal microscope (LSM 780, Zeiss, Germany). Cells were fixed in 4% formaldehyde for 10 min at room temperature followed by permeated with 0.1% Triton-X 100 (Sigma) in 1× PBS for 5 min. Subsequently, the cells were blocked in 1% bovine serum albumin/PBS for 20 min at room temperature. Rabbit monoclonal anti-mouse vinculin (Vcl) antibody (ab129002, Abcam, Cambridge, UK) was added at a 1:200 dilution and incubated at 4 °C overnight, followed by three rinses with PBS. The secondary antibody Anti-rabbit IgG (H + L), F(ab′)2 Fragment conjugated with Alexa Fluor 488 (#4412, Cell Signaling, Carlsbad, CA, US) was added at a 1:1000 dilution and incubated for 60 min. Cells were then incubated with DAPI (Sigma) for 3 min and observed.

The Vcl protein expression was quantified by quantitative real-time PCR (qPCR). Cells were seeded (*n* = 3 per group) in 24-wells plates at a density of 2 × 10^5^ cells/well and incubated at 5% CO_2_ and 37 °C for 1 and 3 days. Primer sequences are shown in Table 2.

Cell adhesion at 3 h was analyzed using a Cell Counting Kit-8 (CCK-8) (Jingxin, Canton, China). Cells at a density of 2 × 10^5^ cells/well were suspended in 1 mL α-MEM and seeded (*n* = 3 per group) in 24-well plates. Cells were rinsed thrice with PBS and then incubated at 37 °C for 2 h according to the manufacture’s instruction of CCK-8 assay. The absorbance was noted at 450 nm by a microplate reader.

#### Cell proliferation

Cells at a density of 2 × 10^4^ cells/well were added onto Ti disks (*n* = 6 per group) and cultured for 3, 5, and 7 days. At each time point, samples were rinsed thrice with PBS and then incubated at 37 °C for 2 h according to the manufacture’s instruction of CCK-8 assay. The absorbance was noted as shown above.

#### Cell differentiation

Cells were seeded at a density of 2 × 10^5^ cells/well on porous and dense Ti disks in 24-well plates in triplicate. After 3 days of incubation, cell differentiation was induced by 50 mg/mL of ascorbic acid and 10 mM β-glycerophosphate and analyzed at the time point of day 1, 3, 7, and 14. The total RNA was isolated by Trizol (Invitrogen, Carlsbad, CA, US) according to the manufacturer’s instructions. Total RNA (2 μg) was reverse transcribed for cDNA using a PrimeScript RT reagent kit (TaKaRa, Kusatsu, Japan). The qPCR was performed thrice with three samples per group by SYBR Premix Ex Taq II (TaKaRa) on the CFX96 RT-PCR System (Bio-rad, Hercules, CA, US). The primers of target genes Vcl, runt-related transcription factor 2 (Runx2), alkaline phosphatase (ALP), osteocalcin (OCN), and bone morphogenetic protein 2 (BMP2), as well as housekeeping gene β-actin, were listed in Table 1.

**Table 1:** Primers used in qRT-PCR

Gene	Forward primer sequence (5′–3′)	Reverse primer sequence(5′–3′)
Vcl	TGGCACATCTGACCTACTGC	TGGTGAGTCAACTCCTGCTG
Runx2	GCCGGGAATGATGAGAACTA	GGACCGTCCACTGTCACTTT
ALP	AACCCAGACACAAGCATTCC	GCCTTTGAGGTTTTTGGTCA
OCN	TTCTGCTCACTCTGCTGACC	ACCACTCCAGCACAACTCCT
BMP2	TCCCCAGTGACGAGTTTCTC	GTCGAAGCTCTCCCACTGAC
β-actin	GCTCTTTTCCAGCCTTCCTT	GTGCTAGGAGCCAGAGCAGT

Cells were seeded at a density of 2 × 10^4^ cells/well on the Ti disks with three disks per group for the ALP activity test. After 7 and 14 days of culture, samples were washed with PBS and 100 μL of 1% Triton X-100 was added to each well. Cells on the disks were stored at 4 °C overnight and added with p-Nitrophenyl Phosphate Substrate (Jiancheng, Nanjing, China). The absorbance of the supernatant was noted as protein absorption in the study.

#### Alizarin red S staining

Cells at a density of 2 × 10^4^ cells/well were seeded on Ti disks in 24-wells plates. After 14 and 21 days of culture, cells were fixed with 4% formaldehyde and washed with PBS. Samples were stained in 2% Alizarin red S solution (pH = 7.2) for 15 min followed by washing thrice with deionized water. The calcified nodules were observed under a stereomicroscope (M205A, Leica, Wetzlar, Germany).

### Statistics

Differences between groups and control were analyzed with one-way ANOVA followed by LSD-*t* test. Factorial design ANOVA was adopted to analyze the main effect and interaction between pore size and porosity. Statistical analyses were performed using SPSS 21.0 (IBM, New York). Differences and parameters were considered statistically significant at a level of 0.05.

## Results

### Structure of porous Ti

Values of nominal porosity and pore sizes of porous Ti samples were calculated (Table 2). The mean porosity (A and B) and pore size (I, II, and III) were adjusted by a mass fraction and particle size of NH_4_HCO_3_. A positive correlation was found between the quantity of NH_4_HCO_3_ and porosity, and also between the size of NH_4_HCO_3_ and pore sizes. The dense Ti group (Group C) acted as controls.

**Table 2:** Porous Ti samples fabricated by using different powder mixtures

Group	NH_4_HCO_3._ (wt%)	NH_4_HCO_3_ (µm)	Porosity (%) (*x* ± sd, sic passim)	Pore size (µm) (*x* ± sd)
AI	20	0–200	43.1 ± 0.7	154.8 ± 11.9
AII	20	200–400	40.9 ± 1.5	295.6 ± 8.5
AIII	20	400–600	44.3 ± 1.1	560.4 ± 25.6
BI	30	0–200	53.3 ± 1.2	191.6 ± 3.7
BII	30	200–400	51.7 ± 2.7	303.8 ± 8.2
BIII	30	400–600	49.9 ± 3.9	583.1 ± 21.7
C	0	0	0	0

**Fig. 1:**
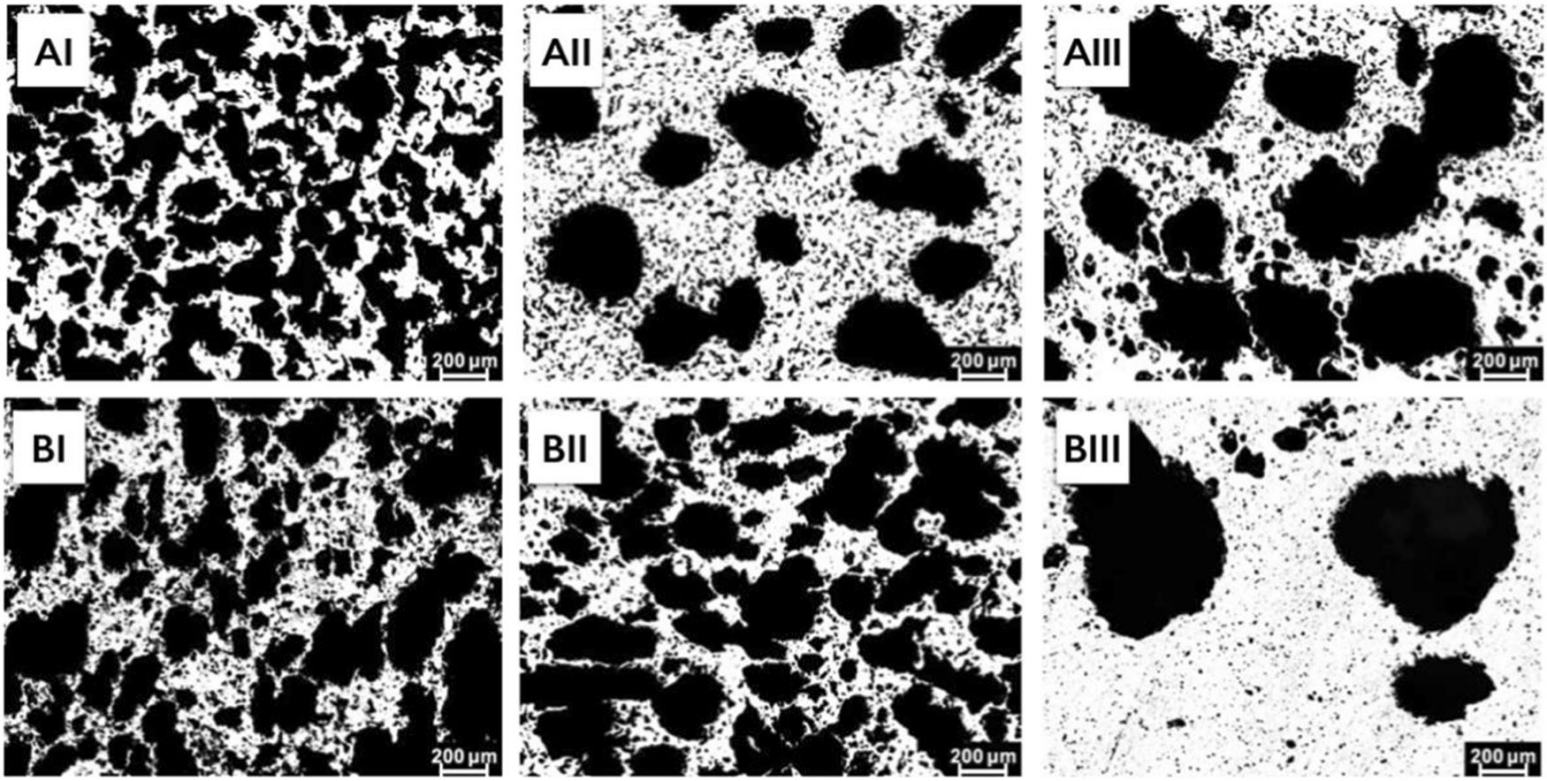
Metallographic top views of porous Ti samples presented in Table 2

**Fig. 2:**
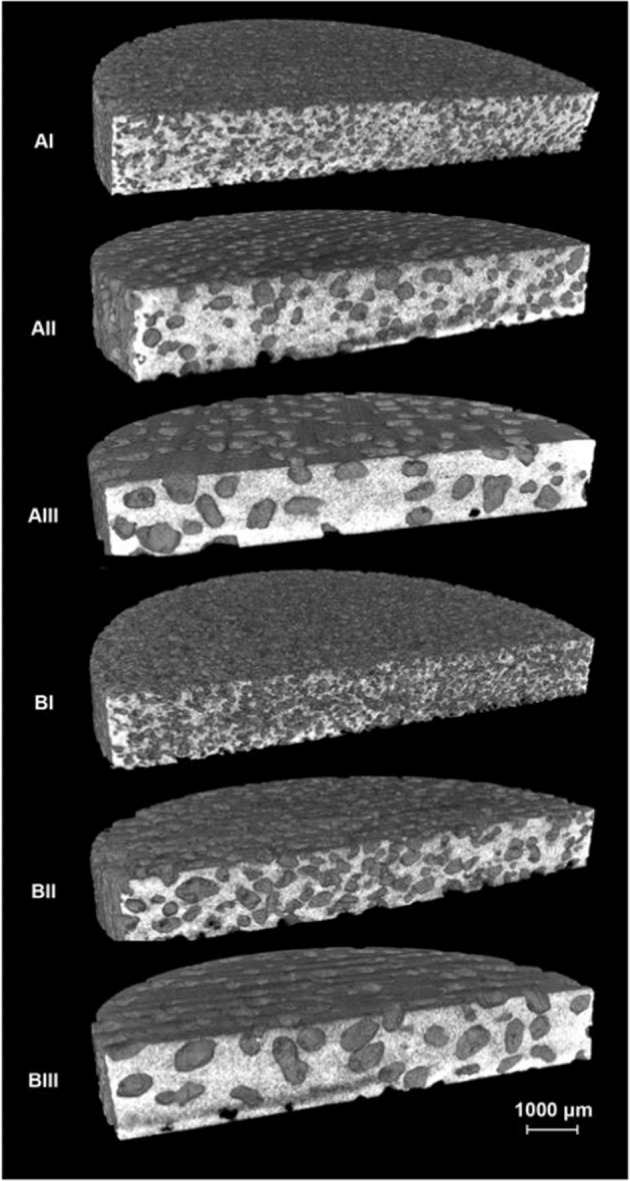
3D reconstruction of porous Ti disc samples

The elongated, square, and blunt pores were homogeneously distributed in all group samples and no cracks or defects were detected (Fig. 1). Open and interconnected pores were observed in Group AI, AII, BI, and BII, especially in Group BI, while pores were mostly isolated in Group AIII and BIII (Fig. 2).

### Mechanical properties

**Fig. 3:**
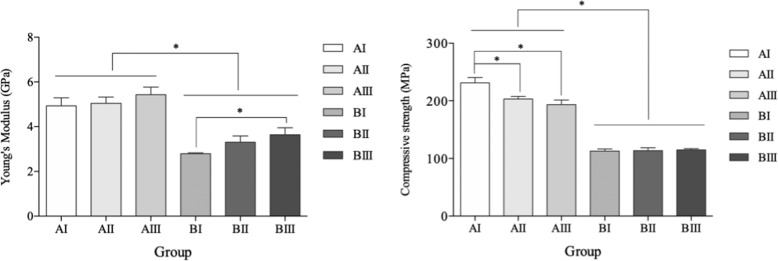
Young’s modulus and compressive strength of porous Ti. * represented *P* < 0.05

It can be seen from the data in Table 3 and in Fig. 3 that porous Ti samples with lower porosity exhibit higher Young’s modulus and compressive strength. No differences were found among Group AI, AII, and AIII with regard to Young’s modulus, and no significant correlation was identified in Group B with different pore sizes in compressive strength. Group BI presented the lowest Young’s modulus of 2.80 ± 0.03 GPa, while Group AI presented the highest compressive strength of 231.1 ± 9.4 MPa. These results show a strong influence of porosity and pore size on the comprehensive mechanical properties of porous Ti.

**Table 3:** Results of compression tests

Group	Compressive strength (MPa)	Young’s modulus (GPa)
AI	231.1 ± 9.4	4.93 ± 0.36
AII	203.1 ± 4.6	5.05 ± 0.28
AIII	193.4 ± 7.9	5.43 ± 0.34
BI	112.4 ± 3.6	2.80 ± 0.03
BII	113.3 ± 5.2	3.30 ± 0.28
BIII	114.5 ± 2.4	3.64 ± 0.31

### Protein adsorption

**Fig. 4:**
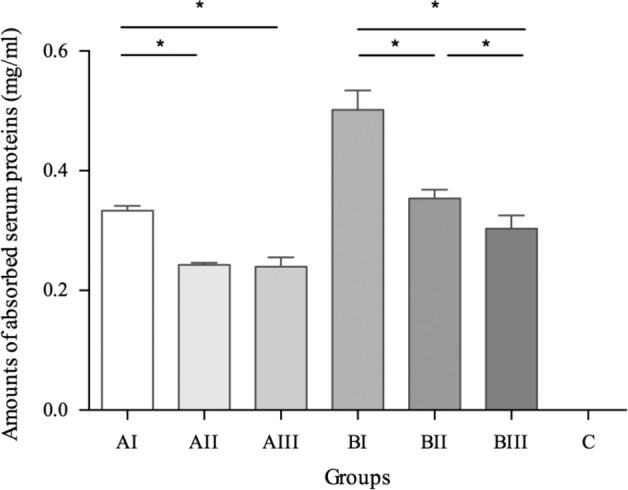
Amount of absorbed total serum proteins on the disks as AI > AII, AI > AIII (*P* = 0.000) and BI > BII > BIII (*P* = 0.000)

Generally, the porous sample adsorbed more protein than controls (*P* = 0.000). Pore size negatively interacted with porosity (*P* = 0.000). Porous Ti under the same mean pore size tended to adsorb more proteins with increasing porosity (*P* = 0.000). Samples with the smallest pore size, and also higher porosity, were favorable to the protein adsorption (*P* = 0.000, Fig. 4).

### In-vitro tests

#### Cell attachment

**Fig. 5:**
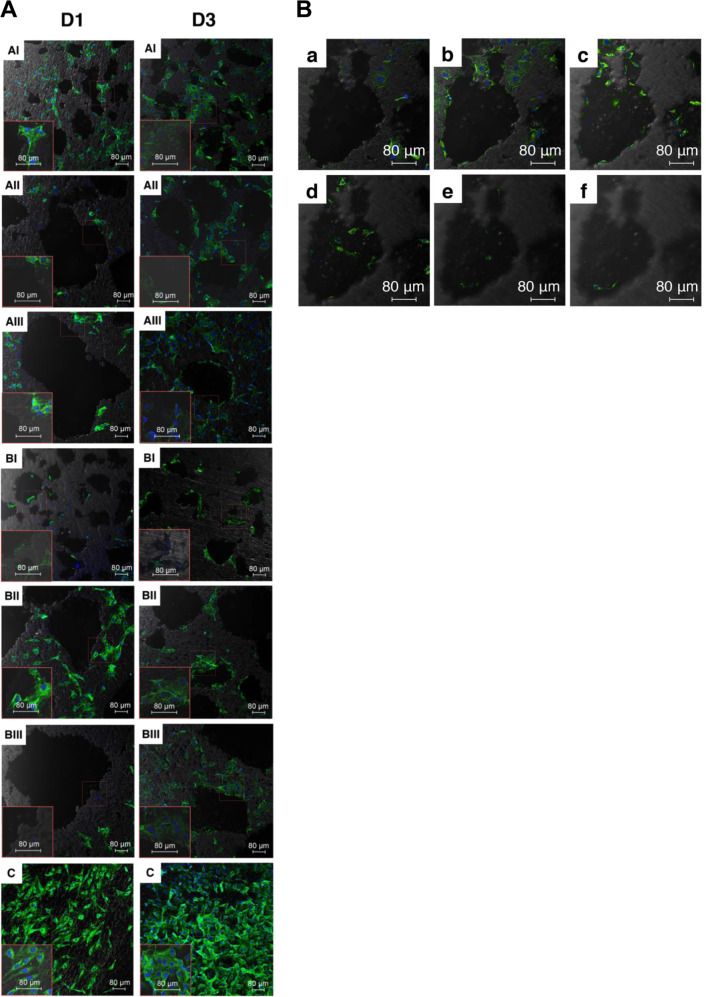
**A** Adhesion of cells labeling Vcl (green) and nucleus (blue) on Ti samples after 1 day and 3 days culture. **B** Cells labeling Vcl (green) after 3 days culture on Group AI. Cells adhesion extended from the sample surface to the pore bottom of the superficial layer (total depth 25.79 μm)

Cells labeling Vcl (green) were well developed and highly organized on the superficial layer of porous Ti with the smallest pore size, yet the attached cells were more evident on dense disks (Fig. 5A). Cells showed a more elongated shape on dense Ti, while major cells presented a less spread shape on porous Ti. Dot-like or dash-like Vcl adhesion sites distributed throughout the cells on all groups.

**Fig. 6:**
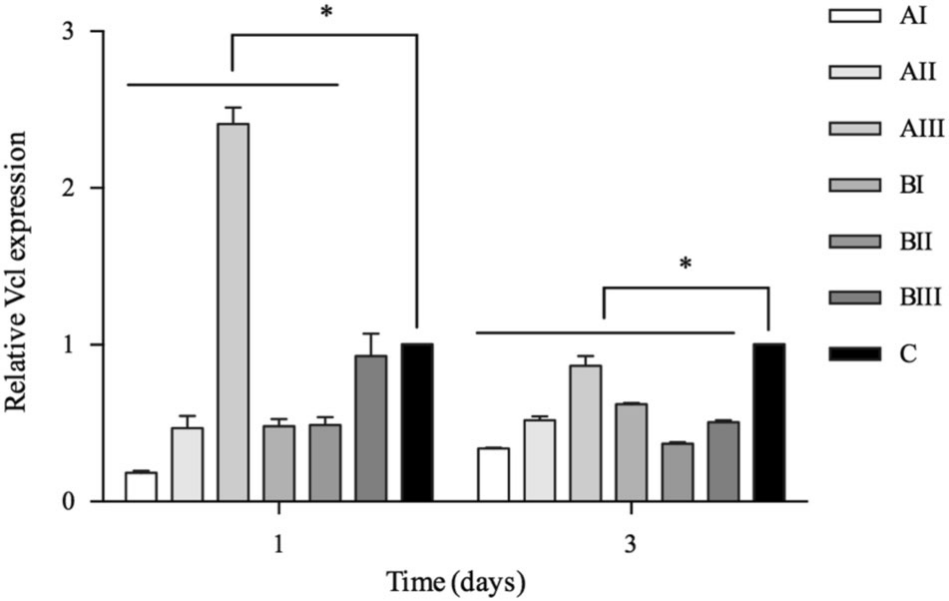
Expression of *Vcl* in cells after 1 and 3 days culture. *Represented *P* < 0.05

The expression of *Vcl* on samples of small pore size (group AI and BI) began already in day 1 culture and kept rising during the 3 days culture compared with the control (*P* = 0.000), whereas the tendency was not found in other groups (Fig. 6).

**Fig. 7:**
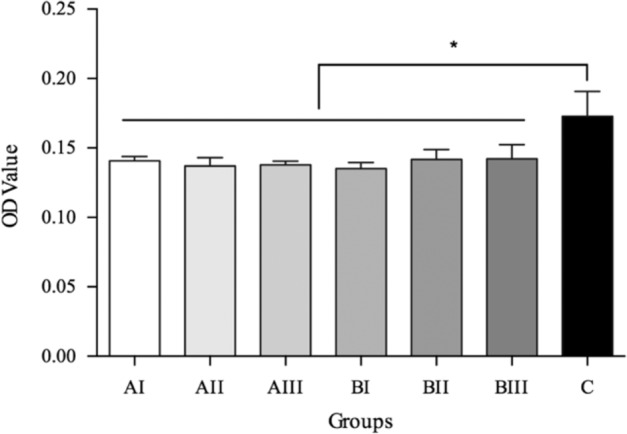
Cell attachment onto the samples after 3 h culture. *Represented *P* < 0.05

Definitely more cells adhered onto the dense Ti sample than onto any other porous ones already after 3 h culture (*P* = 0.001, Fig. 7). No difference was identified within the porous Ti groups (porosity *P* = 0.666; pore size *P* = 0.837).

#### Cell proliferation

**Fig. 8:**
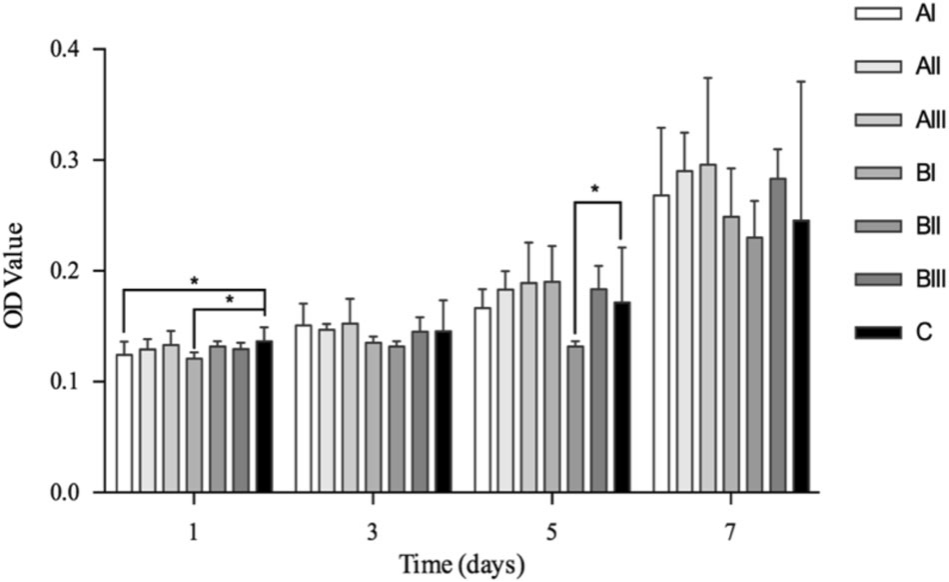
Cell proliferation after 1, 3, 5, and 7 days culture. *Represented *P* < 0.05

Cell proliferation on the porous and dense samples did not seem to be distinguishable from each other (Fig. 8, *P* > 0.05), except for the porous samples with the smallest pore size (AI and BI) on day 1 (*P* = 0.032 and *P* = 0.007, respectively). Cell expanded with time.

#### Osteogenic differentiation

**Fig. 9:**
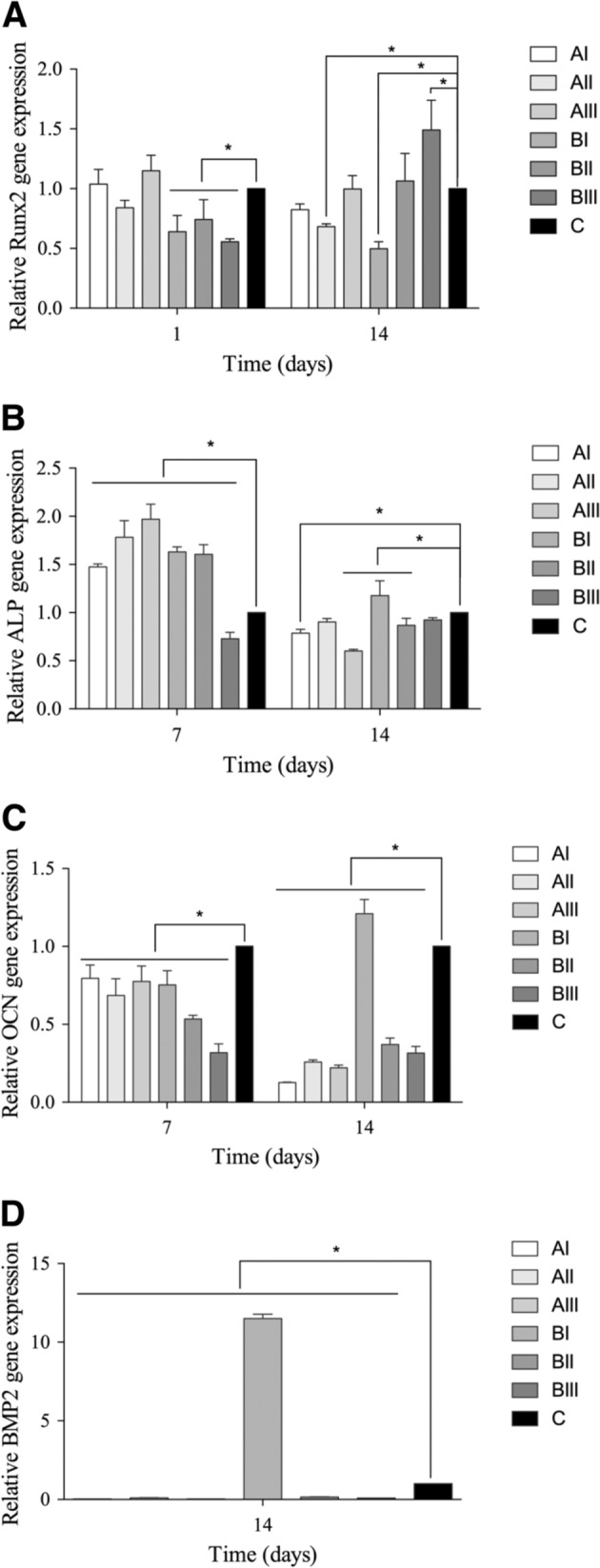
Osteogenic differentiation of cells on porous and dense Ti samples, (**A**) *Runx2*, (**B**) *ALP*, (**C**) *OCN*, (**D**) *BMP2*. *Represented *P* < 0.05

Only those porous samples with the highest porosity and relatively small pore size (group BI) showed more *ALP*, *OCN*, and *BMP2* expression after two weeks of culture (*P* < 0.05, Fig. 9B-D), and the lowest expression of *Runx2* among all groups (*P* = 0.001).

**Fig. 10:**
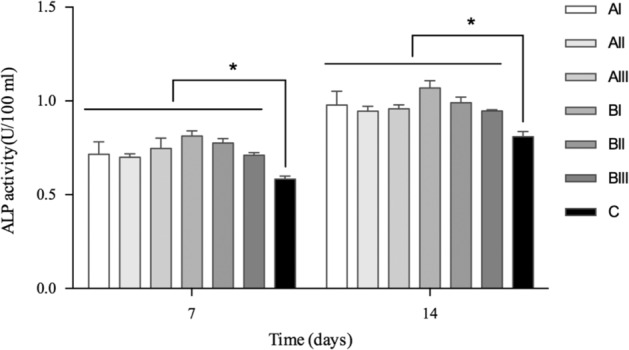
Cell s ALP activity after 7 and 14 days culture. *P < 0.05

Porous samples with the highest porosity and small pore size at the same time (BI) also seemed to be more favorable to ALP enzyme and the early osteoblast differentiation (*P* < 0.05, Fig. 10).

#### Alizarin red S staining

**Fig. 11:**
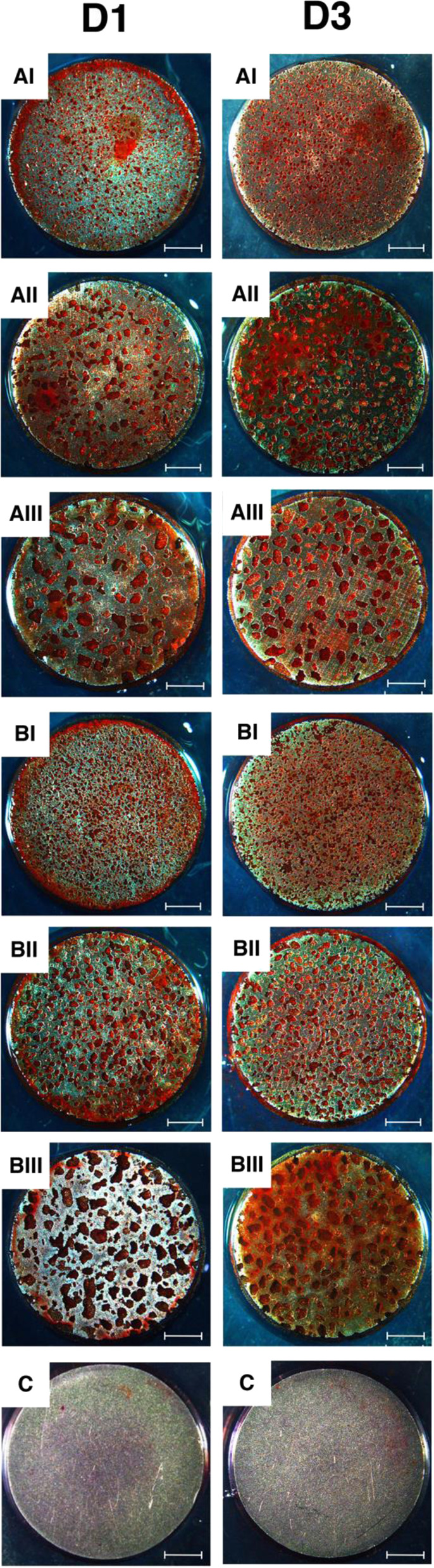
Formation of mineralization nodules. Scale bars: 2 mm

Mineralized nodules were found everywhere on the surface as well as inside the pores in all porous samples, whereas no such nodules could be detected on the dense samples (Fig. 11). The nodules seemed to deposit more inside the pores than on the surface.

## Discussion

It has been well known that higher porosity facilitates bone ingrowth, but weakens the mechanical properties of the Ti implant. Yet a consensus has not been reached with respect to the concrete value of porosity. Since bone formation by osteogenic cells is characterized by protein adhesion, cell proliferation, expression of osteogenic relative markers, and mineralization [19–21], we tried to find out the optimal porosity according to these parameters.

Extensive research has shown that mismatched Young’s modulus between the implant and the bone can cause a severe stress shielding effect on the bone [2]. The Young’s modulus of Group BI (2.80 ± 0.03 GPa) was within the range of human trabecular bone (0.01-3 GPa) and lower than cortical bone (10-30 GPa) which may help to reduce stress shielding effect to reduce bone resorption after implantation [22, 23]. The compressive strength of Group BI (112.4 ± 3.6 MPa) successfully satisfied basic mechanical property requirements [24]. A recent study also confirmed that the space-holder method and the use of NH_4_HCO_3_ as a space holder can reach a biomechanical balance by controlling pore size and porosity separately [25].

Porosity and pore size were twin factors and, as expected, both negatively correlated with each other in the present results. Porous Ti tended to adsorb more proteins with increasing porosity. Porous Ti samples (group BI) with the highest porosity of 53.3% were favorable to the protein adsorption. Higher porosity means more porous spaces in the porous sample and can provide more surface area and anchor for the protein in the superficial layer [26]. Since the first issue of implantation is protein adsorption on the implant surface [27], more protein adsorption will facilitate the subsequent biological process and cell osteogenic differentiation [28, 29].

In the literature, the discussion on the role of pore size remains no consensus. In the present results, the porous samples with the highest porosity also had smaller pore sizes. Porosity, based upon the definition, is only related to material density. A porous structure may reduce the density of dense material and provide certain space by a given material density in two ways: either more pores with smaller pore size or few pores with larger pore size. Higher porosity of porous material is not necessarily linked to smaller pore size [30]. Whether the certain pore size is favorable to the cytocompatibility just as the related porosity can thus only be answered by concrete experiments.

In the present results, samples of small pore size as group AI (154.8 µm) and group BI (191.6 µm) showed the increasing tendency of *Vcl* expression after a 3 days culture. The highest amount of cells adhered to was found in the dense samples. If the dense Ti sample could be treated as a porous sample of “infinitesimal” pore size, porous Ti of small pore size seemed to be favorable to the cell adhesion. In our results, samples of small pore size also had the highest porosity in their own grouping, 43.1%, and 53.3% respectively. Since Vcl is required for focal adhesions assembly [31], and cells on the porous samples showed a flat and well-spread shape indicating strong focal adhesion, it suggested that the combination of higher porosity and small pore size of porous Ti seemed to have better efficacy for cell adhesion.

A previous study has found large pore size was favorable to cell adhesion [32], yet it was conducted on the collagen-based material. In our present study, the cells adhered more to the samples of larger pores (Group AIII 154.8 μm), but only for the first day of culture. After a couple of days of growth, the adhered cell amount decreased, whereas only those samples of small pore size (AI and BI) showed an increasing trend. The introduction of porous structure in the superficial layer yielded more surface area, and the pores of small pore size in the present study accommodated still enough space for cell growth. The slight restriction of cell extending on the porous Ti with small pore size in the initial stage was deemed to be related to the topography of porous Ti, which impairs the interaction between cell and material [33]. In comparison with the result on the dense Ti, it suggested that a porous sample that provided a more flat surface should be in favor of cell attachment.

After cell attachment, cell proliferation and differentiation were the concern. In our results, cell proliferation on the porous and dense samples generally did not seem to be distinguishable from each other. The finding is in accordance with the previous studies on porous bone scaffolds which also found cell proliferation is not affected by pore size [34]. A similar phenomenon was reported on porous Ti that no difference could be identified between pore sizes of 313 μm and 390 μm in cell proliferation. The slightly lower cell proliferation in the 188 μm pore size group was contributed to lower permeability [35].

A porous Ti implant can only be in use by which the interface of implant and bone has good osseointegration. The expression of *Runx2*, *ALP*, *OCN*, and *BMP2* is the key marker of osteoblast differentiation [31, 36, 37]. In our results, only those porous samples with the highest porosity and relatively small pore size (group BI) showed more *ALP*, more *OCN*, and more *BMP2* expression after a 2 weeks culture. It was consistent with the study in which porous material of 188 μm pore size showed higher osteogenic differentiation [35]. Group BI had a pore size of 191.6 µm which was very close to the value of 200 μm suitable for the bone ingrowth that other studies found [38]. In the result, we also found a porous sample of small pore size had the lowest expression of *Runx2* among all groups. Since *Runx2* is the transcriptional factor that initiates bone formation [36], which suggested that small pore size was not beneficial to the osteo-initiation at the very beginning.

In the late cellular calcification, calcium nodules were found everywhere in porous groups but not in the dense ones, suggesting that the introduction of porous structure, independent of the porosity and pore size, was at least better for osseointegration than the dense material.

## Conclusion

Porous Ti with a porosity of 53.3 ± 1.2% and an average pore size of 191.6 ± 3.7 μm fabricated by powder metallurgy process showed the expected mechanical property and improved osseointegration as implants in dental treatment.
